# Comparative Evaluation of the Effect of Brushing Simulation on the Surface Roughness of Acrylic- and Silicone-Based Soft Liners Using Two Different Commercially Available Denture Cleansers: An In Vitro Study

**DOI:** 10.7759/cureus.98426

**Published:** 2025-12-04

**Authors:** Vishakha Sachani, Jyoti B Parihar, Akshita Chipper, Reema Srichand, Swapnali Mhatre, Mridula Joshi

**Affiliations:** 1 Prosthodontics and Crown and Bridge and Implantology, Bharati Vidyapeeth (Deemed to be University) Dental College and Hospital, Navi Mumbai, IND

**Keywords:** acrylic liner, brushing simulation, denture cleanser, present in vitro study, silicone liner, soft denture liners, surface roughness

## Abstract

Objective

This present in vitro study evaluates the impact of brushing simulation on the surface roughness of acrylic-based and silicone-based soft denture liners following immersion in two different denture cleansing solutions.

Materials and methods

Specimens of silicone-based and acrylic-based soft liners were fabricated and subjected to a standardized brushing simulation protocol. Soft liners were suspended into the standard template of 40 mm x 15 mm x 10 mm dimensions. Two denture cleansers were employed during simulated brushing. The first group was brushed with Stim Clanden denture cleanser, and the second group with Clinsodent denture cleaning powder. Surface roughness (Ra and Rz values) was recorded before and after the simulation using a profilometer. Data were statistically evaluated using Mann-Whitney U and Kruskal-Wallis tests to determine significant differences.

Results

A statistically marked increase in surface roughness was noted in both liner types after brushing simulation. Silicone-based liners exhibited comparatively better resistance to surface deterioration than acrylic-based liners.

Conclusion

Brushing simulation with denture cleansers impacts the surface roughness of soft liners, with acrylic-based liners being more susceptible to surface damage. Silicon-based soft liners proved to be effective and more resistant to surface damage.

## Introduction

Soft denture liners have an important role in enhancing the comfort and function of removable prostheses, mainly in patients with atrophic ridges or sensitive mucosal tissues [1]. These materials, primarily classified into silicone-based and acrylic-based liners, serve to absorb masticatory forces and improve denture fit [2]. However, their inherent porosity and surface characteristics make them vulnerable to plaque accumulation, microbial colonization, and material degradation over time [3]. Regular denture cleansing, involving chemical agents and mechanical brushing, is essential for maintaining oral hygiene and extending the service life of these liners [4,5]. Nonetheless, the interaction between cleansing methods and liner materials may adversely affect surface integrity, particularly surface roughness, which can influence both hygiene and comfort [6]. Brushing with denture cleansers is a common method of maintaining denture hygiene [7]. However, the mechanical action combined with the chemical nature of cleansers may contribute to the degradation of liner surfaces, potentially leading to increased surface roughness and microorganism colonization [8].

Maintaining the hygiene and longevity of denture prostheses is critical for both oral health and patient comfort [9]. Denture cleansers are commonly recommended as part of daily denture care to remove biofilm, stains, and microbial deposits. However, while these agents are effective in cleaning, their prolonged or repeated use may have unintended effects on the physical properties of denture base materials, particularly polymethyl methacrylate (PMMA), which is widely used for fabricating removable dentures [10,11]. Clinsodent denture cleaning powder is a chemically formulated cleansing agent specifically designed to maintain the hygiene and longevity of removable prostheses, such as complete and partial dentures [12]. It contains active oxygen-releasing compounds such as potassium persulfate and sodium perborate, which facilitate effective removal of biofilm, stains, and debris by oxidation [13,14]. This is particularly beneficial in preserving the surface integrity of PMMA, the most commonly used denture base material [15]. The inclusion of enzymes such as proteases further enhances its ability to degrade organic matter, thereby reducing the microbial load, including *Candida *species, which are frequently implicated in denture stomatitis [16]. For optimal hygiene, prosthodontists recommend overnight immersion of dentures in a diluted Clinsodent solution, followed by thorough rinsing and gentle brushing [17]. However, this brushing may lead to surface roughness, resulting in the accumulation of microbial film or plaque on the surface of the denture, which may affect oral hygiene [18].

Stim Clanden denture cleaning products, available in paste form, offer several advantages from a prosthodontic perspective. Among the key benefits is their quick and convenient cleaning action - Stim Clanden tablets effectively remove stains, plaque, and odor-causing debris with just a five-minute soak, which improves patient compliance compared to overnight denture cleansers [19]. The Stim Clanden paste, with its mild abrasives and antimicrobial agents such as triclosan, supports effective daily brushing without causing significant surface wear to the acrylic [20]. These formulations are generally gentle on denture materials and help maintain prosthesis aesthetics and hygiene when used correctly. Additionally, while shorter exposure times reduce the risk of material degradation, overuse or improper brushing technique with the paste could still result in gradual surface roughness or microabrasions [21]. One of the key surface characteristics influenced by chemical agents is surface roughness [1]. Increased surface roughness on denture bases can enhance plaque accumulation, promote microbial colonization (especially *Candida albicans*), and compromise aesthetics and comfort [10]. Various commercial denture cleansers, including effervescent tablets and chemical solutions, differ in composition and pH, potentially leading to varying degrees of surface alteration [22].

A profilometer was used to measure the surface roughness and topography of dental materials, including denture bases or soft liners. Surface roughness is a critical factor in prosthodontics, as it influences plaque accumulation, bacterial adhesion, tissue compatibility, wear resistance, and patient comfort. Profilometers provide quantitative data on the microscopic irregularities of a material’s surface, typically expressed as Ra (average surface roughness) in micrometers (µm) [23]. It is essential to understand the impact of denture cleansers on the surface roughness of denture materials to inform clinical practices and support the improvement of denture cleanser formulations. Therefore, this in-vitro study investigates the impact of simulated brushing using two denture cleansers on the surface roughness of silicone-based and acrylic-based soft denture liners, aiming to assess their durability and clinical suitability under routine hygiene practices.

The primary objective of this study was to evaluate and compare the effect of denture cleansers on the surface roughness of two commercially available soft denture liner materials after simulated brushing. The secondary objective was to assess the change in surface roughness within each material before and after brushing. The working hypothesis was that (i) there would be a significant difference in the post-brushing surface roughness between the two liner materials and (ii) each material would demonstrate a significant increase in surface roughness following brushing. Surface roughness was selected as the primary endpoint because it directly influences plaque retention, hygiene maintenance, and long-term patient comfort.

## Materials and methods

Sample preparation and methodology

**Figure 1:**
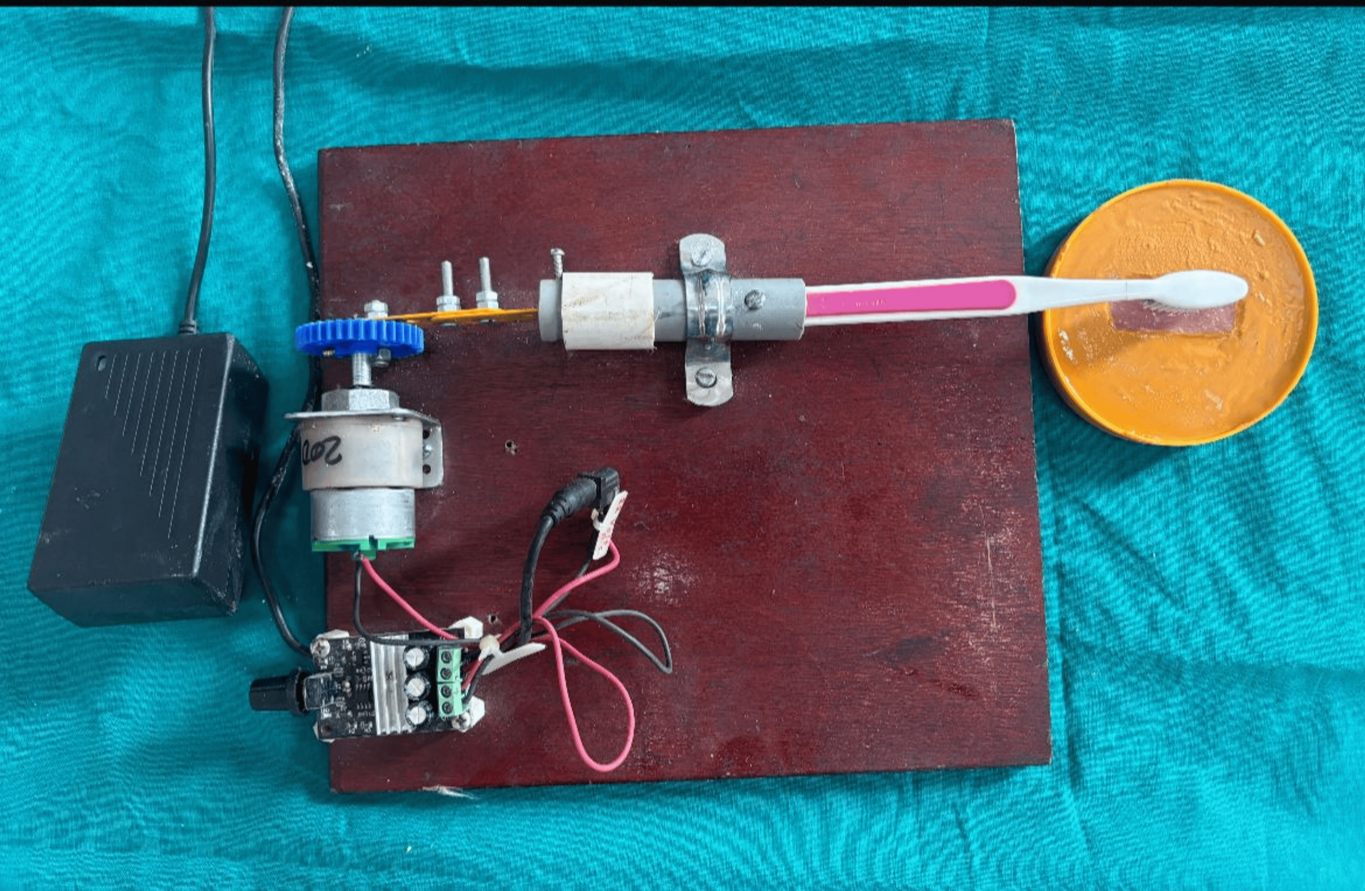
Brushing simulation on the specimens using the tooth brush simulation

**Figure 2:**
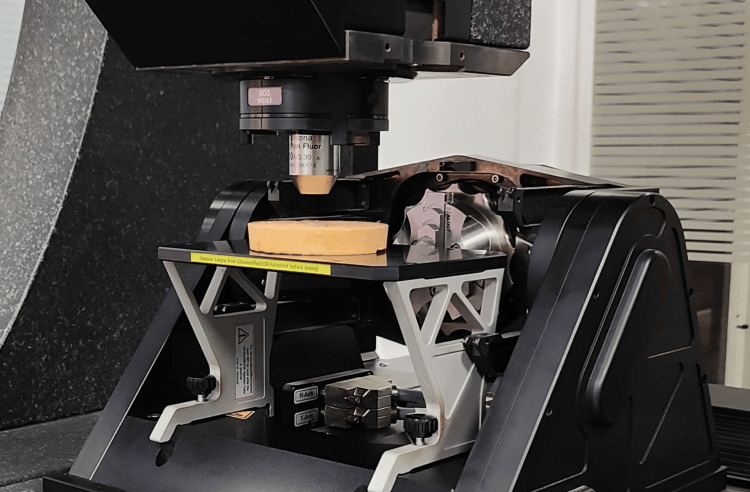
Testing of the sample using a profilometer

A total of 48 rectangular specimens were prepared, of which 24 were made from Acryton soft liner and 24 were made from Reviver soft liner. The required sample size was calculated using the formula for comparing two independent means, with α = 0.05 and 80% power, based on previously reported differences in surface roughness between acrylic and silicone liners after cleanser exposure. Assuming a clinically relevant difference (δ) of 0.20 µm and a standard deviation (σ) of 0.14 µm, the calculation yielded eight specimens per subgroup, resulting in a total sample size of 48. Soft liners were suspended into the standard template of 40 mm x 15 mm x 10 mm dimensions. Standardized rectangular specimens (40 mm × 15 mm × 10 mm) of acrylic-based (Acryton) and silicone-based (Reviver) soft liners were fabricated using stainless steel molds, in accordance with the manufacturers’ instructions. "The specimen dimensions were selected to provide a uniform and standardized soft liner thickness suitable for profilometric assessment, which is consistent with methodologies reported in previous in-vitro studies and falls within the clinically relevant thickness range used for soft denture liners.” After polymerization, all specimens were trimmed to remove excess material and stored in distilled water at 37 ± 1 °C for 24 hours to simulate oral conditions. Distilled water was selected to provide a controlled storage medium without the biochemical and pH variations of natural saliva, thereby maintaining standardization across all specimens and preventing confounding effects during comparison. Each specimen was mounted on a die stone base to provide stability during the brushing simulation, with only the test surface exposed; all other surfaces were covered with wax to ensure uniform mechanical and chemical exposure across samples. The surface roughness of the soft liners was evaluated using a profilometer before simulation (tip 2 µ and 60° angulated). The soft liners were positioned on the die stone bases to enhance stability during the procedure. The liners were brushed with the toothbrush simulator, which mimics manual brushing under standardized conditions. Specimens were fixed with only the test surface exposed, and soft-bristle brushes mounted on reciprocating arms performed X-axis, Y-axis, and circular motions in the presence of the denture cleanser solution, with pre-set motion and stroke frequency to ensure uniform exposure across all samples. The first group was brushed with Stim Clanden denture cleanser, and the second group with Clinsodent denture cleaning powder. A total of 30,000 brushing cycles were performed: 10,000 strokes along the X-axis, 10,000 along the Y-axis, and 5,000 each in clockwise and counterclockwise directions (Figure 1). A total of 30,000 brushing cycles was selected to simulate approximately three years of toothbrushing, based on existing literature where 10,000 cycles represent one year of clinical brushing. After completing the brushing simulation, surface roughness was re-measured using a profilometer (Figure 2), and the results were compared with the baseline values.

Each liner group was divided into subgroups (Table 1). Brushing simulation was done using soft-bristle toothbrushes, which are 0.2 mm in diameter, and a fixed brushing force was used. Brushing was done in the presence of a cleanser solution prepared with distilled water mimicking daily home care. Surface roughness parameters (Ra and Rz) were measured and assessed using a profilometer before and after the brushing simulation. Three readings were taken per sample and averaged.

**Table 1:** Group subdivision for denture cleansers

Type of liner	Groups
Group 1 - Acrylic-based soft liner (Acryton)	Group 1a - Acrylic-based soft liner (Acryton) without denture cleanser
	Group 1b - Acrylic-based soft liner (Acryton) with Stim Clanden denture cleanser
	Group 1c - Acrylic-based soft liner (Acryton) with Clinsodent denture cleaning powder
Group 2 - Silicone-based soft liner (Reviver)	Group 2a - Silicone-based soft liner (Reviver) without denture cleanser
	Group 2b - Silicone-based soft liner (Reviver) with Stim Clanden denture cleanser
	Group 2c - Silicone-based soft liner (Reviver) with Clinsodent denture cleaning powder

Statistical analysis

Statistical analysis was performed using the Mann-Whitney U test and the Kruskal-Wallis test to evaluate intergroup differences. A p-value of less than 0.05 was considered statistically significant.

## Results

The control group exhibited no significant changes in surface roughness following the brushing simulation. In contrast, specimens with acrylic-based soft liners demonstrated a marked increase in surface roughness (Ra and Rz), particularly when treated with the alkaline peroxide-based denture cleanser. Silicone-based liners showed comparatively smaller increases in roughness, suggesting greater resilience to mechanical abrasion and chemical exposure. All statistical analyses were performed using Statistical Product and Service Solutions (SPSS, version 21.0; IBM SPSS Statistics for Windows, Armonk, NY). The surface roughness measurements (Ra, expressed in micrometers) were reported as mean ± standard deviation.

Before conducting comparative analyses, a normality test was performed to evaluate the distribution of the data. The Mann-Whitney U test was applied, and the results indicated that the data were normally distributed across all groups, as evidenced by p-values greater than 0.05. This confirmed the suitability of using parametric statistical tests for further analysis. For group comparisons, both the Mann-Whitney U test and the Kruskal-Wallis test were utilized to examine the impact of two independent variables: the type of liner material (silicone-based or acrylic-based) and the cleansing method employed (control, Clinsodent, or Stim Clanden). The primary outcome measure was the change in surface roughness (ΔRa), calculated by subtracting the baseline roughness values from those recorded after the brushing simulation for each specimen. These statistical tests were conducted to determine differences in surface roughness changes among the groups and to determine the significance of the observed effects.

The analysis revealed significant main effects of both material type and cleanser type, as well as an interaction effect between these factors. Regarding material type, acrylic-based liners exhibited a significantly greater increase in surface roughness (ΔRa) compared to silicone-based liners across all cleansing protocols (p < 0.05). In terms of cleanser type, specimens treated with Clinsodent demonstrated a higher alteration in surface roughness relative to those cleaned with Stim Clanden, indicating that Stim Clanden may be the preferable option for maintaining the surface integrity of soft liners. Nonetheless, it is important to note that both cleansing agents contributed to some degree of roughness change (p < 0.01). Additionally, a significant interaction was observed between the liner material and the type of cleanser used (p < 0.05), suggesting that the abrasive effects of the cleansers varied depending on the specific liner material.

There was a statistically highly significant difference seen (p<0.01) for Ra B within the acrylic-based soft liner (I) and silicone-based soft liner (II) groups, with higher values in the acrylic-based soft liner (I) group; the surface roughness after brushing (Ra A) between the acrylic-based soft liner (I) and silicone-based soft liner (II) groups, with higher values in the acrylic-based soft liner (I) group; and the average surface roughness (Rz) diff between the acrylic-based soft liner (I) and silicone-based soft liner (II) groups, with higher values in the acrylic-based soft liner (I) group. There was a statistically significant difference seen (p<0.05) for Rz B between the acrylic-based soft liner (I) and silicone-based soft liner (II) groups, with higher values in the acrylic-based soft liner (I) group; and Rz A between the acrylic-based soft liner (I) and silicone-based soft liner (II) groups, with higher values in the acrylic-based soft liner (I) group. There was a statistically non-significant difference seen (p>0.05) for Ra diff of the acrylic-based soft liner (I) and silicone-based soft liner (II) groups (Table 2).

**Table 2:** Inter-group pair-wise comparison of Group I (Acryton) vs II (Reviver) for Ra B, Ra A, Ra diff, and Rz B, Rz A, and Rz diff using the Mann-Whitney U test Ra: Roughness average, Rz: Peak and depth average value Mann-Whitney U test; **indicates statistically highly significant p<0.01; * indicates statistically significant p<0.05; # indicates statistically non-significant p>0.05.

Variables	Group	N	Mean	Std. Deviation	Mean Rank	Sum of ranks	Median	Mann-Whitney U value	Z value	p value of Mann-Whitney U test
Ra B	I	24	3.66096	1.033018	30.56	733.5	3.99	142.5	-3	0.003**
	II	24	2.99458	1.335379	18.44	442.5	2.97			
Ra A	I	24	3.92571	1.023344	31.1	746.5	4.24	129.5	-3.268	0.001**
	II	24	3.15171	1.325701	17.9	429.5	3.16			
Ra diff	I	24	0.26475	0.223784	28.15	675.5	0.19	200.5	-1.804	0.071#
	II	24	0.15712	0.088213	20.85	500.5	0.12			
Rz B	I	24	30.87842	7.652921	28.48	683.5	31.18	192.5	-1.969	0.049*
	II	24	25.33404	9.47807	20.52	492.5	21.62			
Rz A	I	24	31.95679	7.87227	28.92	694	32.38	182	-2.186	0.029*
	II	24	25.69471	9.418469	20.08	482	22.5			
Rz diff	I	24	1.07838	0.802167	30.58	734	1.02	142	-3.01	0.003**
	II	24	0.36067	0.32146	18.42	442	0.23			

There was a statistically non-significant difference seen for the values between the subgroups (p>0.05) for all values, while there was a statistically highly significant difference seen for the values between the subgroups (p<0.01) for Ra diff, with a higher value in the acrylic-based soft liner with Clinsodent (Ic) subgroup; Rz diff with a higher value in the acrylic-based soft liner with Clinsodent (Ic) subgroup; and Rz PC diff with a higher value in the acrylic-based soft liner subgroup with Clinsodent (Ic). Meanwhile, there was a statistically significant difference seen for the values between the subgroups (p<0.05) for Ra PC diff, with a higher value in the silicone-based soft liner with Clinsodent (IIc) subgroup, and Ra A, with a higher value in the acrylic-based soft liner with Stim Clanden (Ib) subgroup in acrylic- and silicone-based soft liners (Table 3).

**Table 3:** Inter-group comparison of Ra B, Ra A, Ra diff, Rz B, Rz A, and Rz diff in Ia, Ib, Ic, IIa, IIb, and IIc subgroups Ra: Roughness average, Rz: Peak and depth average value Kruskal-Wallis Test; **indicates statistically highly significant p<0.01; *indicates statistically significant p<0.05; # indicates statistically non-significant p>0.05

Variables	Subgroup	N	Mean	Std. Deviation	Median	Mean Rank	Chi-square value	p-value of the Kruskal-Wallis test
Ra B	Ia	8	3.62275	1.295704	3.96	30.88	9.757	0.082#
	Ib	8	3.495	1.135849	3.99	29.25		
	Ic	8	3.86513	0.675637	3.81	31.56		
	IIa	8	3.32625	2.207038	2.69	21.63		
	IIb	8	2.83788	0.706792	2.86	17.31		
	IIc	8	2.81963	0.5476	2.97	16.38		
Ra A	Ia	8	3.73713	1.304404	4.06	29.19	11.893	0.036*
	Ib	8	3.7675	1.009848	4.3	29.25		
	Ic	8	4.2725	0.716205	4.14	34.88		
	IIa	8	3.41125	2.216614	2.82	20.25		
	IIb	8	3.00987	0.672249	3.06	16.69		
	IIc	8	3.034	0.541739	3.16	16.75		
Ra diff	Ia	8	0.11437	0.042551	0.12	15.44	21.827	0.001**
	Ib	8	0.2725	0.181345	0.22	31.88		
	Ic	8	0.40738	0.284059	0.27	37.13		
	IIa	8	0.085	0.041297	0.09	9.69		
	IIb	8	0.172	0.076487	0.16	24		
	IIc	8	0.21437	0.089824	0.22	28.88		
Rz B	Ia	8	29.75338	13.029803	29.25	25.63	8.351	0.138#
	Ib	8	31.43375	3.460965	30.91	29.31		
	Ic	8	31.44812	2.917383	31.95	30.5		
	IIa	8	27.82438	13.205319	26.97	24.81		
	IIb	8	20.37213	6.533641	19.7	12.5		
	IIc	8	27.80563	5.99102	29.39	24.25		
Rz A	Ia	8	30.07162	13.338902	29.29	24.5	9.976	0.076#
	Ib	8	32.47725	3.198544	32.16	29.5		
	Ic	8	33.3215	2.994625	33.37	32.75		
	IIa	8	28.02825	13.11877	27.13	24.63		
	IIb	8	20.89063	6.530003	20.3	12.25		
	IIc	8	28.16525	6.095042	29.89	23.38		
Rz diff	Ia	8	0.31825	0.440258	0.19	13.88	29.839	0.000**
	Ib	8	1.0435	0.37463	0.99	34.75		
	Ic	8	1.87338	0.629327	1.88	43.13		
	IIa	8	0.20388	0.171008	0.14	12.63		
	IIb	8	0.5185	0.437145	0.4	22.88		
	IIc	8	0.35962	0.250254	0.22	19.75		

## Discussion

This in vitro investigation was designed to assess and compare how simulated brushing with two denture cleansers, Clinsodent and Stim Clanden, affects the surface roughness of soft denture liners made from silicone and acrylic materials. Surface roughness is a crucial factor in maintaining oral prosthetic hygiene because greater roughness can encourage biofilm buildup, diminish patient comfort, and shorten the functional lifespan of soft liners [10].

These liners are composed of substances belonging to different chemical families. With continuous use, they gradually undergo chemical transformations due to contact with saliva in the mouth or immersion in water and denture cleansers when stored [24]. The most frequently encountered issues related to using denture cleansers include hardening of the liner, increased porosity, absorption of odors, uptake of water, dissolution of material, changes in color, and the growth of bacteria and fungi [25]. The hardness of a denture liner is a critical property because it directly affects its malleability, ductility, and resistance to wear. In this context, hardness represents the material’s ability to resist permanent surface indentation or penetration, which in turn influences its adaptability and durability in the oral environment. Adequate malleability allows the liner to deform slightly under load and adapt to the denture-bearing tissues without cracking, while sufficient ductility ensures it can withstand tensile and functional stresses without tearing. Optimal hardness also provides resistance to wear from mastication, parafunctional habits, and denture cleaning procedures. If the liner is too hard, it may lose its cushioning effect and cause mucosal trauma; if it is too soft, it may abrade rapidly, leading to increased surface roughness and greater susceptibility to plaque accumulation and microbial colonization. Additionally, surface roughness plays an essential role, as a rougher surface can promote biofilm accumulation and facilitate the colonization of *C. albicans *[26]. Our findings demonstrated that both cleansers, when used in conjunction with brushing simulation, resulted in a statistically significant increase in surface roughness across both types of soft liners. However, the magnitude of surface change varied depending on the type of denture liner and the cleanser used. Silicone-based liners generally exhibited greater resistance to surface degradation compared to acrylic-based liners, which can be attributed to their inherent chemical stability - derived from strong silicon-oxygen (Si-O) bonds, hydrophobic methyl side groups that limit water absorption, and a crosslinked network that resists swelling and chemical attack - whereas the weaker carbon-carbon (C-C) bonds and higher porosity of acrylic-based liners make them more susceptible to plasticizer leaching and surface roughening under mechanical and chemical exposure, which may better withstand the mechanical and chemical effects of brushing and cleanser exposure. Acrylic-based liners, being more porous and hydrophilic, appeared more susceptible to surface roughening. The plasticizers in acrylic-based materials may leach out under the combined effects of cleanser chemistry and mechanical abrasion, leading to surface deterioration; in contrast, silicone-based liners derive most of their flexibility from the inherent elasticity of the Si-O backbone but may also contain silicone-compatible oils or low-molecular-weight siloxanes as plasticizers. These additives are more chemically compatible with the polydimethylsiloxane (PDMS) matrix and less soluble in water, making them less prone to rapid leaching. However, prolonged exposure to oxidizing cleansers or elevated temperatures can still cause gradual loss or migration of these silicone oils, resulting in slight hardening or minor surface dullness over time. Although this effect is less pronounced than in acrylic-based liners, it may still contribute to surface changes during long-term clinical use [14].

Effective control of denture plaque is critical for preserving oral hygiene and preventing associated infections in individuals wearing removable prostheses. Traditionally, plaque removal has relied on mechanical methods such as brushing. However, these approaches may not be suitable for all denture types, particularly those incorporating soft denture liners - due to the risk of surface degradation and material distortion. As a result, chemical cleansing agents, including commercially available denture cleansers, are often recommended as the primary method for maintaining hygiene in such cases. These cleansers provide a non-abrasive alternative that can effectively reduce microbial load while preserving the physical properties of resilient lining material [27].

When two denture cleansers were compared, Clinsodent resulted in a lesser increase in surface roughness, indicating a relatively milder interaction with the denture liner materials. Stim Clanden, on the other hand, caused a greater increase in surface roughness, particularly with the acrylic-based liner. These results are consistent with previous studies, such as Huddar et al. [28] and Yuzugullu [29], that highlight differences in how various cleanser formulations interact with prosthetic materials. The brushing simulation further amplified the mechanical stress on the liner surfaces, simulating the cumulative effect of daily oral hygiene practices. Acrylic-based dentures, which are the most commonly used type, are made from polymethyl methacrylate (PMMA) and require specific cleansers that can penetrate the material’s porous surface without causing abrasion or structural damage [18]. Cleansers designed for acrylic dentures typically come in the form of effervescent tablets or soaking solutions and contain ingredients such as sodium bicarbonate, sodium perborate, and enzymatic agents. These components help in breaking down organic matter, removing stains, and controlling microbial growth, especially targeting pathogens such as *C. albicans *and *Streptococcus mutans* [12].

On the other hand, silicone-based denture materials, often used in soft liners and flexible prosthetics, present a different set of challenges. These materials are more hydrophilic and pliable, making them susceptible to microbial colonization and degradation when exposed to strong chemicals [30]. As a result, silicone-based denture cleansers are formulated to be gentler, avoiding oxidizing agents and harsh acids that could compromise the material’s elasticity and comfort. They often include mild surfactants and antimicrobial agents such as chlorhexidine or cetylpyridinium chloride to clean effectively without altering the physical properties of the silicone. Using the correct type of cleanser based on the denture material is crucial, as inappropriate products can lead to surface damage, reduced functional lifespan of the denture, and increased risk of oral infections. Therefore, when selecting a denture cleanser, both the material composition and the user’s specific oral health needs must be taken into account [31,32]. For patients using soft liners, especially those with compromised oral hygiene or mucosal sensitivity, silicone-based liners in combination with a milder cleanser such as Clinsodent may be preferable. Brushing should be advised with caution or supplemented by soaking methods alone in cases where mechanical abrasion exacerbates surface degradation.

Clinsodent and Stim Clanden cleansers result in a measurable increase in surface roughness (Ra) for both acrylic-based and silicone-based soft denture liners. Notably, the magnitude of change varied by both liner type and cleanser used - acrylic liners consistently exhibited greater roughening compared to their silicone counterparts. This aligns with earlier research showing that acrylic liners are more susceptible to surface deterioration due to cleanser exposure: Mohammed et al. observed significant Ra increases in acrylic liners treated with alkaline peroxide cleansers compared to minimal changes in silicone liners [33]. Tan et al. also reported that perborate-containing agents leach plasticizer from silicone materials, contributing to surface alterations [34]. Comparative studies reinforce these trends. For example, Handa et al. found that alkaline peroxide cleansers increased Ra in acrylic liners significantly more than in silicone liners across multiple time points [35]. Furthermore, García et al. and Brożek et al. in 2003 and 2011, respectively, noted that, while certain cleansers left silicone liners relatively unaffected, acrylic materials displayed marked increases in roughness and hardness over time [2,36].

In our brushing simulation, Clinsodent - an alkaline peroxide powder - induced greater roughness than Stim tablets, especially in acrylic liners. This may stem from Clinsodent’s higher alkalinity and oxygen release, which can aggressively degrade polymer chains or leach plasticizers, leading to surface irregularities and micro-porosity. Stim Clanden, being milder, generated less pronounced but still clinically relevant changes [17]. Clinically, an elevated surface roughness is concerning because rougher denture liner surfaces have a greater tendency to retain dental plaque, food debris, and microorganisms - particularly *C. albicans*. When the surface irregularities exceed about 0.2 µm Ra, microbial adhesion increases significantly, which can predispose patients to denture stomatitis, halitosis, and inflammation of the underlying mucosa. Increased roughness can also compromise the aesthetics of the denture, make it harder for patients to clean effectively, and accelerate material degradation through wear and staining. Apart from mechanical and chemical cleaning methods, the composition of the denture liner significantly influences surface roughness. Silicone-based liners, with strong Si-O bonds, hydrophobic methyl groups, and higher crosslink density, absorb less water and leach fewer components, helping maintain a smooth surface. This dense, hydrophobic network means cleansers can act mainly on the outer surface and, in most cases, penetrate without causing abrasion or structural damage [34-36]. In contrast, acrylic-based liners (PMMA with plasticizers) are more hydrophilic and porous, allowing deeper penetration of cleanser solutions, which can aid in biofilm removal but also increase the risk of plasticizer loss, micro-abrasion, and surface deterioration [9,11,14,25,35]. The presence and bonding quality of inorganic fillers [6,7], as well as crosslink density [2,36], also influence how the liner responds to cleanser exposure, further affecting roughness over time. Over time, this can reduce the functional lifespan of the prosthesis and negatively impact patient comfort and oral health. Profilometric and microbiologic evidence confirms that increases in Ra beyond 0.2 µm heighten microbial retention - particularly *Candida *spp. - predisposing to denture stomatitis and odor. Although silicone liners proved more resistant, even these materials are not immune to cumulative surface damage with recurrent abrasive cleaning and chemical exposure.

Limitations and future directions

Although the present study was conducted under standardized in vitro conditions, the absence of dynamic intraoral variables - including temperature cycling, salivary enzymes, variable pH, and masticatory load - may have influenced the extent of surface roughness change observed. Soft liners may deteriorate differently in vivo as a result of mechanical deformation, saliva buffering action, and microbial colonization, which were not simulated in the current design. Furthermore, although the sample size was statistically adequate for detecting differences in surface roughness, a larger sample size or inclusion of multiple liner brands and cleanser formulations could potentially reveal subtler interaction effects. These limitations suggest that the degree of surface roughness change reported in this study should be interpreted as reflective of controlled laboratory conditions rather than a complete replication of the clinical environment.

## Conclusions

Brushing simulation in combination with denture cleansers was observed to increase the surface roughness of soft denture liners, with acrylic-based materials being more susceptible to these changes than silicone-based liners. Among the cleansers evaluated, Stim Clanden demonstrated comparatively lower abrasiveness, suggesting it may be preferable for maintaining liner surface integrity. Overall, Reviver proved more effective than Acryton in preserving the smoothness of the denture liners. Additionally, acrylic soft liners exhibited the greatest roughness increase when brushed with Clinsodent cleanser, followed by Stim Clanden.

Based on these findings, future formulations of denture cleansers could be improved by reducing abrasive components, lowering alkalinity, and optimizing the concentration of active agents to minimize surface degradation while retaining cleaning efficacy. Additionally, incorporating biocompatible enzymes and gentler surfactants may offer better long-term outcomes, especially for soft liner materials.
